# Long-term results comparison after anterior cervical discectomy with BGS-7 spacer (NOVOMAX®-C) and allograft spacer: A prospective observational study

**DOI:** 10.3389/fbioe.2023.1100462

**Published:** 2023-04-19

**Authors:** Seungjun Ryu, Dal-Sung Ryu, Keun-Su Kim

**Affiliations:** ^1^ National Health Insurance Service Ilsan Hospital, Goyang, Republic of Korea; ^2^ Department of Neurosurgery, School of Medicine, Eulji University, Daejeon, Republic of Korea; ^3^ School of Medicine, Inha University, Inchon, Republic of Korea; ^4^ Gangnam Severance Hospital, Seoul, Seoul, Republic of Korea

**Keywords:** bioactive glass-ceramic, cervical fusion, long-term clinical outcome, allograft, finite element analysis

## Abstract

**Introduction:** In an anterior cervical discectomy and fusion (ACDF), various types of graft materials including autograft, allograft, and synthetic graft have been used to achieve adequate spinal fusion. Allograft spacer is mainly used in cervical fusion, especially in the anterior approach. The synthetic bone graft material BGS-7(CaO-SiO2-P2O5-B2O3, bioactive Glass-Ceramics) can bind with surrounding bone tissue by forming a hydroxyapatite layer bone bridge, leading to faster graft osseointegration. This study was conducted to compare long-term clinical outcome of BGS-7 spacer and allograft spacer for anterior cervical discectomy and fusion surgery.

**Materials and Methods:** From September 2014 to December 2016, Consecutive anterior cervical discectomy and fusion surgeries using a BGS-7 spacer (N = 18) and Allograft spacer (N = 26) were compared for postoperative clinical outcomes. Radiologic assessments were performed, and Instrumental failure, including breakage, cage migration, subsidence were observed and Fusion status were analyzed. Finite element analysis was performed for simulating mechanical stress between the vertebral body and implant. Clinical outcomes were evaluated using neck VAS, NDI, and JOA on the patient’s final follow-up visits.

**Results:** Among the 44 patients who underwent an anterior cervical discectomy and fusion surgery using the BGS-7 spacer and Allograft spacer, there were 30 men and 14 women. The average age at the operation was 47.69 ± 10.49 in allograft spacer and 51.67 ± 11.03 in BGS-7 spacer. The mean follow-up period was 89.18 ± 5.44 months. Twenty three (88.46%) patients in allograft spacer and 20(100%) patients in BGS-7 spacer were demonstrated radiologic evidence of interbody fusion in last OPD, which accounts for fusion grade 4 or 5. Peak stresses were 343.85 MPa in allograft spacer, and 132.55 MPa in BGS-7 spacer. Long-term clinical outcomes including neck VAS, NDI, and JOA didn’t show statistical differences between the two groups. There were no adverse events related to the BGS-7 spacer.10.3389/fbioe.2023.110046.

**Conclusion:** The BGS-7 spacer demonstrated reliability as a spacer in anterior cervical discectomy and fusionF surgery without instrumental failure. Early stabilization with a bony bridge formation was observed at the intermediate follow-up period, and the long-term clinical outcome was favorable at more than 60 months after surgery without any adverse events. Thus, the BGS-7 spacer is a safe and effective alternative to the allograft spacer in anterior cervical discectomy and fusion surgery.

## Introduction

In an anterior cervical discectomy and fusion (ACDF) surgery, Allograft cervical spacer is the second most used material after autogenous bone because of its many advantages, such as decreased operation time, low donor site morbidity, and stable bone union rate. (Wigfield and Nelson, 2001). However. the use of an allograft spacer in ACDF has a risk of occurring various complications including infection or immune reaction due to donor tissue as well as cage-related complications including subsidence, cage migration, breakage, and collapse. (Woo et al., 2019; Menon et al., 2021; Yang et al., 2021). Several bone substitutes have been developed to overcome such limitations, including bioactive ceramics, beta-tricalcium phosphate, and hydroxyapatite. (Ilharreborde et al., 2008). Those materials have been replacing an autograft spacer in ACDF surgery nowadays.

Titanium has shown high strength and biocompatibility, but it requires filler material and does not fuse with adjacent bone tissue. In addition, the subsidence rate is high, and the metal artifact on an MRI is profound. A polyether ether ketone (PEEK) cage is the most widely used spacer due to its reasonable strength, biocompatibility, and low subsidence rate; however, it also requires filler material and does not fuse with the bone. Hydroxyapatite (HA) has several advantages, including acceptable biocompatibility, osteoconductivity, a low subsidence rate, and fusion ability with bone. (Lee et al., 2013a). But the clinical application was not successful because a breakage rate after insertion was frequently reported, which ranged from 2 to 49%. (Suetsuna et al., 2001; Ito et al., 2002; McConnell et al., 2003; Xie et al., 2006; Falavigna et al., 2009).

BGS-7 has been reported to promote the osteoblastic differentiation of human mesenchymal stem cells (Lee et al., 2013a). *In vivo* model, the dense cylindrical shaped specimen demonstrated better bone bonding to adjacent bones compared with hydroxyapatite (Lee et al., 2010a), and it was found to stimulate osseointegration of implants when coating the surface (Lee et al., 2011; Lee et al., 2013b). In addition, BGS-7 didn’t show toxicity and adverse effects in a repetitive intravenous toxicity study (Lee et al., 2010b). Nevertheless, its chronic and long-term toxicity and adverse effects were still unknown.

BGS-7 was a commercialized bio-synthetic intervertebral fusion material in 2014; however, there was no report about long-term clinical outcomes after ACDF surgery with BGS-7. This is the first study to compare the long-term clinical outcome of BGS-7 spacer (NOVOMAX®-C) and allograft spacer (CERVICAL SPACER C+), including finite element analysis for biomechanical properties of each implant.

## Materials and methods

### Study design

From September 2014 to December 2016, Consecutive ACDF surgeries (N = 44) using a BGS-7 spacer (NOVOMAX®-C, CGBio Co., Ltd., Seoul, Republic of Korea) or allograft spacer (Cervical spacer C+, CGBio Co., Ltd., Seoul, Republic of Korea) were performed in a single medical center by a single expert surgeon. We compared fusion rate and clinical outcomes between the two groups of patients at more than 60 months after surgery. The disease entity was a degenerative cervical disease from the C3/4 to C6/7 levels, including cervical disc herniation and foraminal stenosis. The use of patient data for research purposes was approved by the institutional review boards (IRB No. 3-2021-0437**)**, and the patients provided written informed consent.

The inclusion criteria were 1) adult men and women over the age of 19, 2) patients with bioactive ceramic spacer or allobone for cervical anterior intervertebral disc removal in single or multiple segments fusion from September 2014 to December 2016, 3) a patient who voluntarily signed a written consent form. The exclusion criteria included 1) patients who voluntarily did not agree to participate in the study, 2) Patients with other factors influencing clinical trial product effectiveness assessment.

### Surgical technique

The surgical techniques followed a standard ACDF surgery procedure, which includes a plate augmented interbody fusion, using a BGS-7 spacer or allograft. Linear anterior incisions were made on the opposite side of the main neural compression. For example, if the patient had a left-side protrusion at C5/6, the incision was made on the right side. The Smith-Robinson anterior cervical approach was employed to expose the anterior surface of the cervical disc. The disc material was removed with a No 15. blade and forceps. Meticulous curettage was performed to remove the cartilage portion of the upper and lower endplate, leaving the bony structure intact. The uncinate process was removed with a high-speed drill to obtain a wide decompression of the foraminal space, if needed. The posterior longitudinal ligament was resected only when it was needed for decompression, such as for the removal of a ruptured disc material that exited through the ligament. After confirmation of a complete neural decompression, a proper sized BGS-7 spacer or allograft was impacted into the empty disc space. Plate augmentation was applied for all patient’s entire surgical levels.

### Clinical outcome measurement

Radiologic assessment was performed using a cervical spine X-ray series at the preoperative, immediate postoperative, and long term follow-up time points. A breakage of the cage was defined as the presence of a crack within the inserted cage or a destructive change to the cage’s shape on the postoperative cervical spine X-ray. Subsidence was defined as a decrease in interbody height of more than 3 mm on the follow-up plain film. The difference of subsidence was based on the length between the center of the upper and lower endplate in a lateral cervical spine X-ray. Fusion status was evaluated by the Brantigan and Steffee fusion grade using an X-ray at the final follow-up visit (Table 1) (Brantigan and Steffee, 1993), and also by stability based inter-spinous motion measurement methods. (Song et al., 2014). The clinical outcome was evaluated using neck visual analogue scale (VAS), neck disability index (NDI) and Japanese Orthopaedic Association (JOA) neck questionnaire on the patient’s follow-up time points.

**TABLE 1:** Fusion result by Brantigan and Steffee (Lee et al., 2010b).

Grade	Classification	Description
1	Obvious pseudarthrosis	Collapse of construct, loss of disc height, vertebral slip, broken screws, displacement of the cage, resorption of bone graft
2	Probable pseudarthrosis	Significant resorption of the bone graft, major lucency, or gap visible in fusion area (2 mm around the entire periphery of graft)
3	Uncertain	Bone graft visible in the fusion area at approximately the density originally achieved at surgery. A small lucency or gap may be visible involving a portion of the fusion area with at least half of the graft area
4	Probable fusion	Probable fusion bone bridges entire fusion area with at least the density achieved at surgery. There should be no lucency between the donor and vertebral bone
5	Obvious fusion	Fusion bone in the fusion area is radiographically more dense and mature than originally achieved by surgery. Optimally, there is no interface between the donor bone and the vertebral bone, although a sclerotic line between the fusion area, resorption of the anterior traction spur, anterior progression of the graft within disc space, and fusion of facet joints

### Finite element analysis

The mechanical performance of the BGS-7 spacer was confirmed using finite element analysis. The comparative model is the allograft spacer. Implant models and a three-dimensional cervical spine vertebral body model were constructed. The region of interest between implants and bone-implant interface was set up using different element sizes that could be distinguished from other parts. The Young’s modulus and Poisson’s ratio were applied to each moiety using previously published data in the literature. (Table 2) (Kumaresan et al., 1999; Kim et al., 2020; Kwak et al., 2021)

**TABLE 2:** Material properties applied to finite element analysis.

Part	Young’s modulus (MPa)	Poisson’s ratio
Cortical bone	10,000	0.29
Cancellous bone	100	0.29
BGS-7 spacer	121,000	0.28
Allograft spacer	17,000	0.30

Two types of surgical models were constructed by placing each implant between the vertebral bodies using Abaqus software. (Hibbett, Karlsson & Sorenson Inc, JOHNSTON, United States) Sliding contact was applied as the boundary condition between the vertebral body and the implant. The inferior endplate of the most caudal vertebra (C6) was fixed in all degrees of freedom, while 1,200N loads were applied to the superior endplate of the most cephalic vertebral level (C6). (Patwardhan et al., 2000). The peak von mises stress (PVMS) and contact pressure that occurs when the same compressive load of 1,200N is applied to the two models are predicted and compared. Information on the number and type of elements used in the FEA study is as follows; element type; tetrahedral mesh (both), number of nodes; 248,215 (BGS-7 spacer) vs. 239,802 (allograft spacer), number of elements; 1,341,817 (BGS-7 spacer) vs. 1,283,606 (allograft spacer). The stress and contact pressure of the vertebral body were used to determine the risk of subsidence, and the stress and contact pressure of the implant were used to evaluate the risk of implant yield.

Stress and contact pressure of the vertebral body were used to determine the risk of subsidence, and the risk of implant yield was confirmed using the stress of the implant.

### Statistical analysis

Descriptive data are represented as mean ± standard error of the means, and statistically compared using two-sample *t*-test or paired *t*-test. For the comparison of categorical variables, chi-square tests and Fisher’s exact tests were performed between two independent groups. All *p*-values less than 0.05 were considered statistically significant. All statistical analyses were performed using SPSS version 26.0 (SPSS Inc., Chicago, IL, United States) by a statistical professional.

## Results

### Demographics

Among the patients who underwent an ACDF surgery using the BGS-7 spacer and Allograft spacer, there were 30 men and 14 women. (Table 3). The average age at the operation was 47.69 ± 10.49 in allograft spacer and 51.67 ± 11.03 in BGS-7 spacer. The mean follow-up period was 89.18 ± 5.44 months. The number of fused segments ranged from a single-level to 3-level. The thirty-four patients had surgery with a 1-level fusion, the nine patients with a 2-level fusion, and one patient with a 3-level fusion.

**TABLE 3:** Demographic data and surgical levels.

	BGS-7 spacer (N = 18)	Allograft spacer (N = 26)	*p*-value
Age (years)	51.67 ± 11.03	47.69 ± 10.49	0.2332^a^
Sex			
Male	12 (66.67)	18 (69.23)	0.8575^b^
Female	6 (33.33)	8 (30.77)	
Fusion level			
1-level	14 (77.78)	20 (76.92)	1.0000^c^
2-level	4 (22.22)	5 (19.23)	
3-level	0 (0.00)	1 (3.85)	
Fusion site	(N = 22)	(N = 33)	0.4397^c^
C3-C4	1 (4.55)	2 (6.06)	
C4-C5	3 (13.64)	10 (30.30)	
C5-C6	13 (59.09)	13 (39.39)	
C6-C7	5 (22.73)	8 (24.24)	
Follow-up period (months)	83.39 ± 2.09	93.19 ± 2.67	**<0.0001** ^a^

^a^ Two-sample *t*-test.

^b^ Chi-square test.

^c^ Fisher’s exact test.

### Radiologic outcome

BGS-7 spacer showed no adverse findings at 60 months after surgery when compared with allograft spacer (Table 4). Average subsidence length was 1.33 ± 0.74 mm per level in BGS-7 spacer group and 2.27 ± 1.39 mm per level in allograft spacer group. Subsidence defined as greater than 3 mm was found in 4 of 18 patients (22.22%) only in the allograft spacer group but not in the BGS-7 spacer group. However, all of them did not complain of significant axial pain and the subsidence rate didn’t increase over follow up period. No breakage was observed like fracture line or deformation of the BGS-7 spacer on X-ray during the whole follow-up period. However, six patients in the allobone spacer group showed breakage of the spacer that resulted in a decreased height rather than the intended disc height.

**TABLE 4:** Radiological outcomes (Complications).

	BGS-7 spacer (N = 18)	Allograft spacer (N = 26)	*p*-value
Subsidence	N = 22	N = 18	
Subsidence rate, N (%)	0 (0.00)	4 (26.67)	**0.0335** ^a^
Subsidence length, mean ± S.D.	1.33 ± 0.74	2.27 ± 1.39	**0.0090** ^b^
Complications	N = 22	N = 33	
Cage breakage, N (%)	0 (0.00)	6 (18.18)	0.0708^a^
Cage migration, N (%)	0 (0.00)	1 (3.03)	1.0000^a^
Instrument failure, N (%)	0 (0.00)	0 (0.00)	1.0000^a^

^a^ Fisher’s exact test.

^b^ Two-sample *t*-test.

**FIGURE 1:**
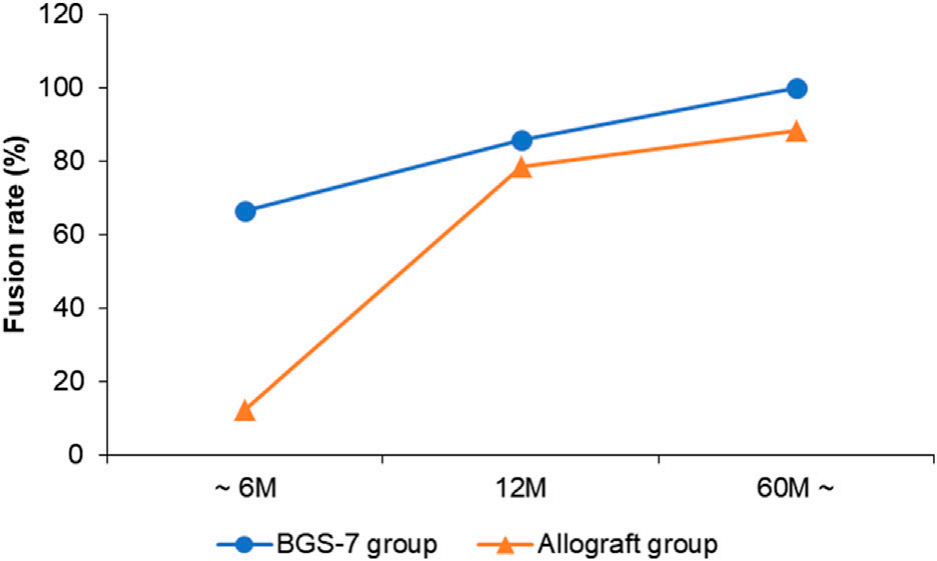
Fusion rate of each group over time.

The result of fusion according to Brantigan and Steffee grading is compared allograft group and BGS-7 group at Table 5 and Figure 1. Patients who were followed for less than 6 months showed stable settlement without early instrumental failure such as cage extrusion. Patients who were followed for 12 months demonstrated a settled fusion progression. Allograft group showed twenty-three of 26 patients who were followed for more than 60 months had satisfactory fusion results with grades 4,5; however, they included 2,1 patients with fusion grade 2, 3, respectively. Twelve patients who were followed for 12 months demonstrated satisfactory fusion result with grades 4, 5 in BGS-7 group. Eighteen of 18 patients who were followed for more than 60 months had enough fusion results with grades 4,5; and there were no patients with fusion grade 2, 3, respectively.

**TABLE 5:** Results of Brantigan and Steffee fusion grading based on CT scan.

	Grade 1	Grade 2	Grade 3	Grade 4	Grade 5	Fusion rate
6M						
Allograft (n = 8)	0 (0.0)	0 (0.0)	7 (87.5)	1 (12.5)	0 (0.0)	1 (12.5)
BGS-7 (n = 6)	0 (0.0)	0 (0.0)	2 (33.3)	4 (66.7)	0 (0.0)	4 (66.7)
*p*-value						0.0909^a^
12M						
Allograft (n = 14)	0 (0.0)	0 (0.0)	3 (21.4)	3 (21.4)	8 (57.1)	11 (78.5)
BGS-7 (n = 14)	0 (0.0)	1 (7.1)	1 (7.1)	5 (35.7)	7 (50.0)	12 (85.7)
*p*-value						1.0000^a^
≥60M						
Allograft (n = 26)	0 (0.0)	2 (7.7)	1 (3.8)	13 (50.0)	10 (38.5)	23 (88.5)
BGS-7 (n = 28)	0 (0.0)	0 (0.0)	0 (0.0)	8 (44.4)	10 (55.6)	18 (100.0)
*p*-value						0.2579^a^

^a^ Fisher’s exact test.

**FIGURE 2:**
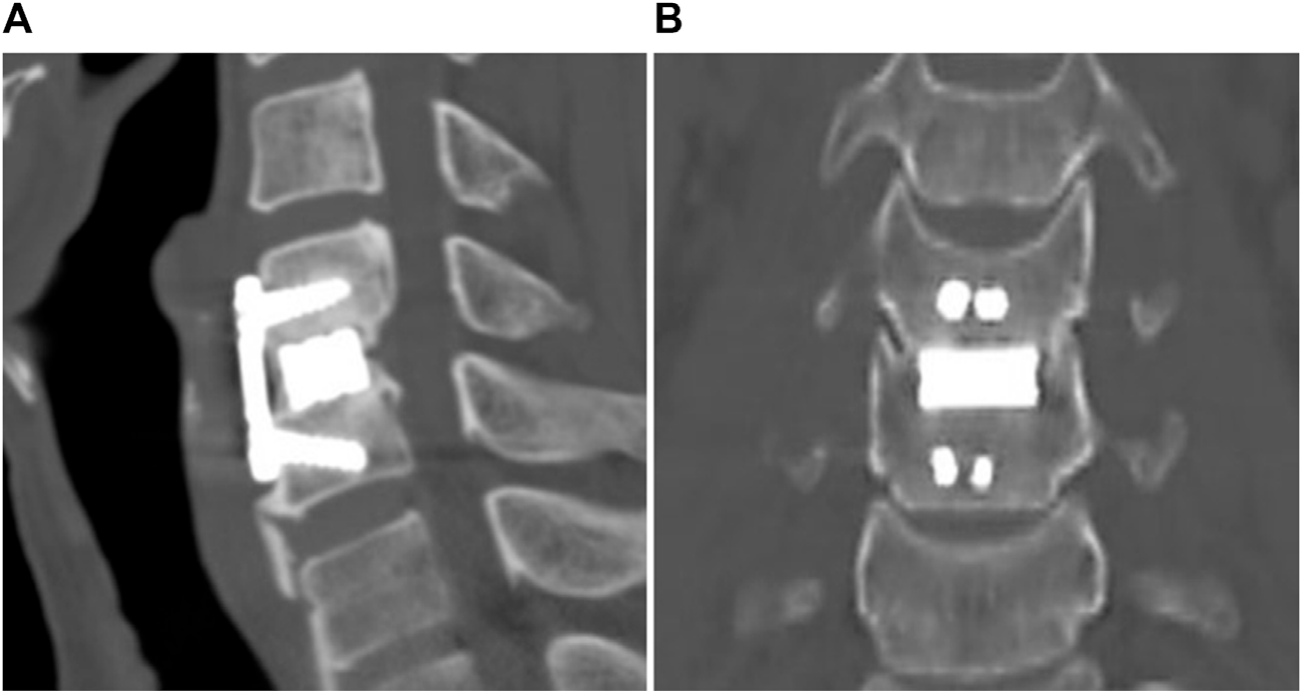
One year post-operative images from CT scan. **(A)** Sagittal view, **(B)** coronal view.

**FIGURE 3:**
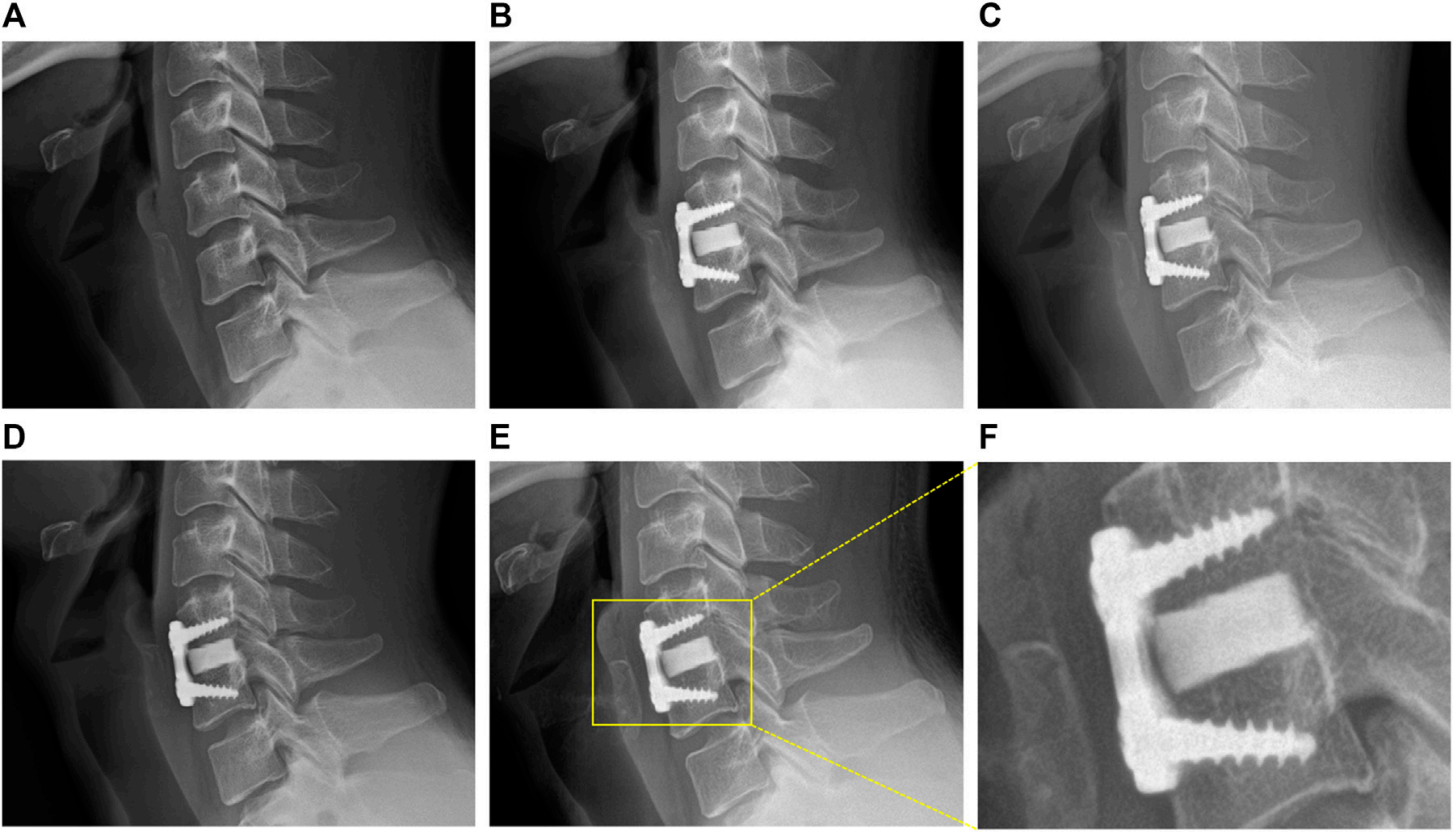
Representative images of BGS-7 spacer-induced segmental fusion progression after ACDF surgery during 5 years. **(A)** Pre-OP, **(B)** 1 month, **(C)** 3 months, **(D)** 1 year, **(E)** present, **(F)** present - magnified.

The sagittal and coronal reconstruction of CT 1 year postoperatively revealed a detailed image of a bony bridge formation, which surrounded the spacer (Figure 2). An example of bioactive glass induced segmental fusion progression after ACDF surgery during 5 years is shown in Figure 3. After 5 years of surgery, it was confirmed that the endplate was in close contact with the spacer boundary, and new bone formation was observed surrounding the spacer (Figure 3).

Table 6 showed pseudoarthrosis evaluation that dynamic stability based fusion status at long-term postoperative period, and there were no statistical differences.

**TABLE 6:** Evaluation of fusion based on dynamic stability (≥60M).

	BGS-7 spacer (N = 18)	Allograft spacer (N = 26)	*p*-value
Mean difference*	0.49 ± 1.37	0.62 ± 1.47	0.7750^a^
Fusion rate			
Fusion	14(77.78)	17(65.38)	0.5069^b^
Non-fusion	4(22.22)	9(34.62)	

*Mean difference = F ISM—E ISM.

^a^ Two-sample *t*-test.

^b^ Chi-square test.

F ISM; cervical flexion inter-spinous distance, E ISM; cervical extension inter-spinous distance; An inter-spinous motion (mean difference) less than 1.0 mm is defined as fusion.

### Clinical outcome

**FIGURE 4:**
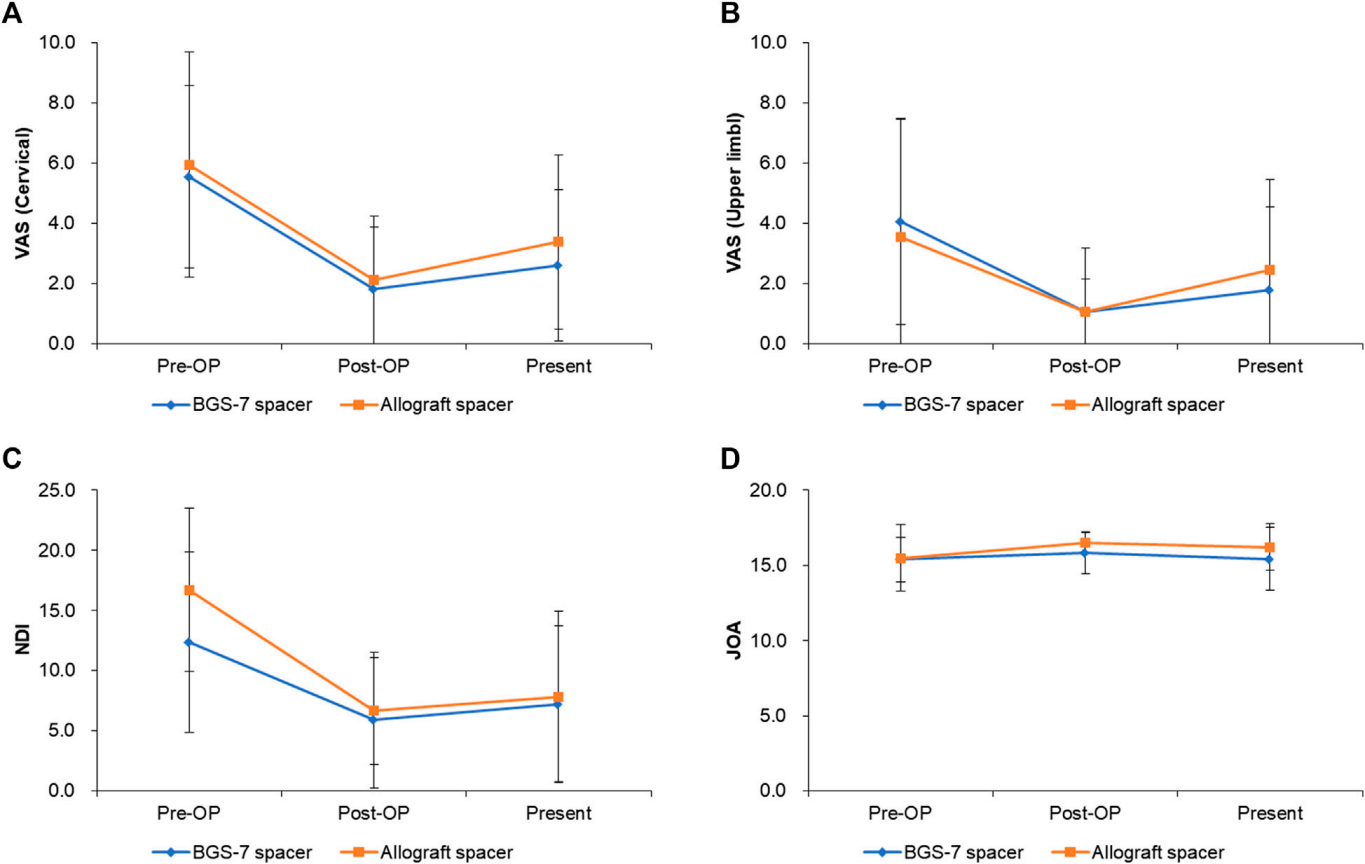
Clinical outcomes for each group after ACDF surgery during 5 years. **(A)** VAS (cervical), **(B)** VAS(upper limb), **(C)** NDI, **(D)** JOA.

The result of clinical outcomes for the BGS-7 group and the allograft group are shown in Table 7 and Figure 4. Visual analogue scale (VAS) for cervical pain showed 5.6 ± 3.0 and 6.0 ± 3.8 at pre-operative status for BGS-7 spacer and allograft spacer, respectively (*p* = 0.706). And the VAS at the immediate postoperative status and long-term postoperative status for BGS-7 spacer and allograft spacer were 1.8 ± 2.0 and 2.1 ± 2.1 (*p* = 0.662), and 2.6 ± 2.5 and 3.4 ± 2.9 (*p* = 0.365), respectively.

**TABLE 7:** Clinical outcomes.

	BGS-7 spacer (N = 18)	Allograft spacer (N = 26)	*p*-value^a^
VAS (Cervical)	N = 18	N = 26	
Pre-op	5.6 ± 0.7	6.0 ± 0.7	0.706
Post-op	1.8 ± 0.5	2.1 ± 0.4	0.662
*p*-value (pre-post)^b^	**<.001**	**<.001**	
Final follow-up	2.6 ± 0.6	3.4 ± 0.6	0.365
*p*-value (pre-final)^b^	**0.001**	**0.010**	
VAS (Upper limb)	N = 18	N = 26	
Pre-op	4.1 ± 0.8	3.5 ± 0.8	0.655
Post-op	1.1 ± 0.3	1.1 ± 0.4	0.968
*p*-value (pre-post)^b^	**0.002**	**0.003**	
Final follow-up	1.8 ± 0.7	2.5 ± 0.6	0.448
*p*-value (pre-final)^b^	**0.029**	0.189	
NDI	N = 18	N = 24	
Pre-op	12.3 ± 1.6	16.7 ± 1.5	0.064
Post-op	5.6 ± 1.1	7.3 ± 1.2	0.309
*p*-value (pre-post)^b^	**0.001**	**<.001**	
Final follow-up	6.9 ± 1.7	9.3 ± 1.3	0.293
*p*-value (pre-final)^b^	**0.035**	**<.001**	
JOA	N = 18	N = 26	
Pre-op	15.4 ± 0.4	15.5 ± 0.4	0.853
Post-op	15.8 ± 0.3	16.5 ± 0.1	**0.037**
*p*-value (pre-post)^b^	**0.042**	**0.018**	
Final follow-up	15.4 ± 0.5	16.2 ± 0.3	0.157
*p*-value (pre-final)^b^	0.911	0.157	

^a^ Two-sample *t*-test.

^b^ Paired *t*-test.

Neck disability index (NDI) showed 12.3 ± 6.8 and 16.7 ± 7.50 at pre-operative status for BGS-7 spacer and allograft spacer, respectively (*p* = 0.064). And the NDI at the immediate postoperative status and long-term postoperative status for BGS-7 spacer and allograft spacer were 5.6 ± 4.4 and 7.3 ± 5.9 (*p* = 0.309), and 6.9 ± 7.1 and 9.3 ± 7.1 (*p* = 0.293), respectively.

Japanese orthopedic association (JOA) score showed 15.4 ± 1.5 and 15.5 ± 2.2 at pre-operative status for BGS-7 spacer and allograft spacer, respectively (*p* = 0.853). And the NDI at the immediate postoperative status and long-term postoperative status for BGS-7 spacer and allograft spacer were 15.8 ± 1.4 and 16.5 ± 0.6 (*p* = 0.037*), and 15.4 ± 2.1 and 16.2 ± 1.6 (*p* = 0.157), respectively.

### Finite element analysis

**FIGURE 5:**
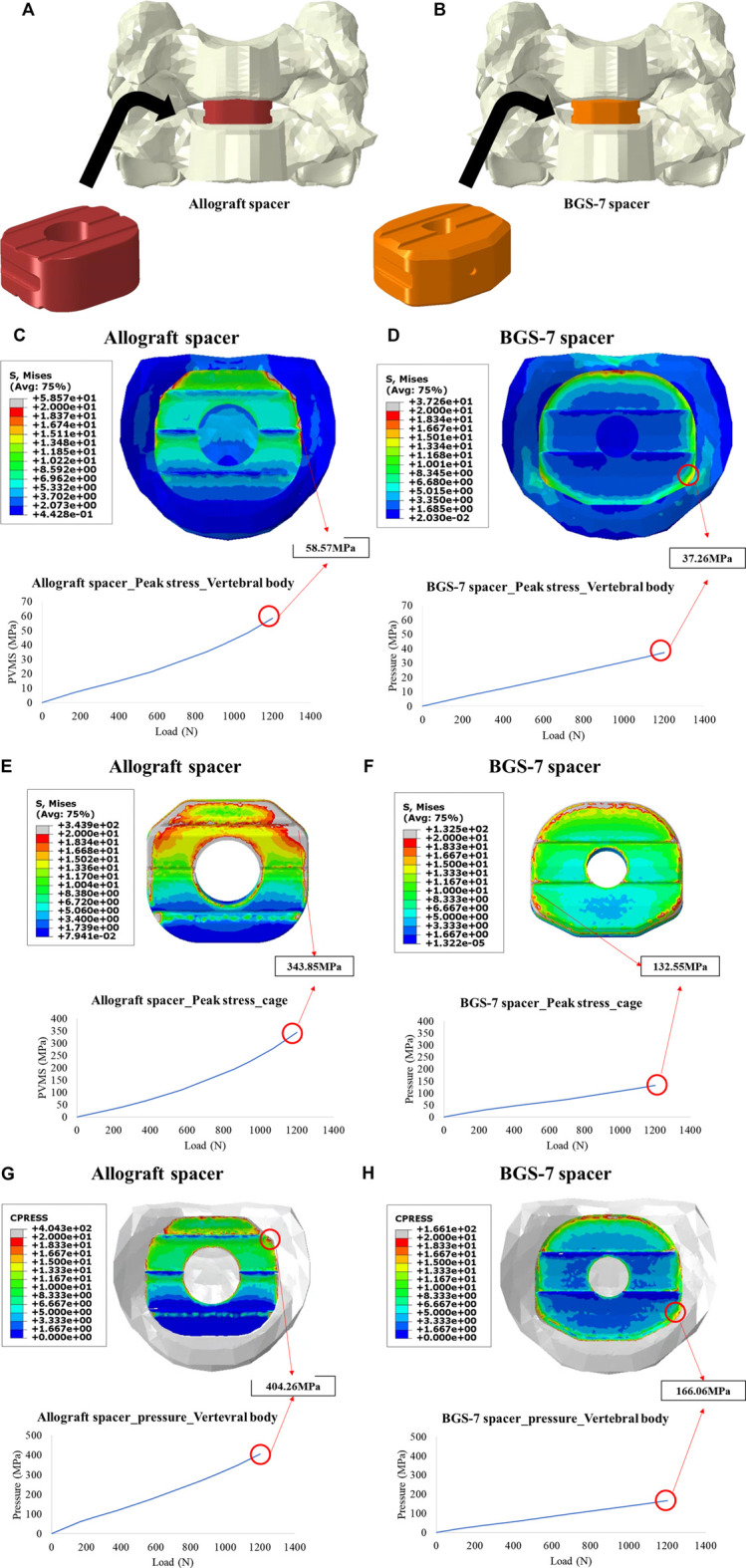
Illustration for two finite element models for the ACDF with allograft spacer **(A)**, and BGS-7 spacer **(B)**. Peak stress on vertebral body under 1200N of compressive load for the allograft spacer **(C)**, and BGS-7 spacer **(D)**. Peak stress on the cage under 1200N of compressive load for the allograft spacer **(E)**, and BGS-7 spacer **(F)**. Contact pressure under 1200N of compressive load for the allograft spacer **(G)**, and BGS-7 spacer **(H)**.

In order to compare the risk of subsidence and implant yield of the allograft spacer and the BGS-7 spacer, the peak von mises stress (PVMS) and contact pressure of the vertebral body/implant were analyzed by finite analysis (Figures 5A, B). The peak von mises stress (PVMS) of the vertebral body due to the compressive load was measured to be about 57% higher the allograft spacer (58.57 MPa) than the BGS-7 spacer (37.26 MPa). (Figures 5C, D) The peak von mises stress generated in the implant is higher in the allograft spacer despite the lower elastic modulus of the material. (Figures 5E, F) The contact pressure of the allograft spacer is higher than that of the BGS-7 spacer, indicating that the risk of subsidence of the BGS-7 spacer is lower than that of allograft spacer. (Figures 5G, H). It can be expected that the allograft spacer has a higher risk of implant yield due to compressive load.

## Discussion

Bioactive ceramics include calcium phosphate compounds (Wigfield and Nelson, 2001; Woo et al., 2019; Menon et al., 2021; Yang et al., 2021) and bioactive glass or glass-ceramics (Ito et al., 2002; Ilharreborde et al., 2008; Lee et al., 2013a). (Lee et al., 2013a) Among the bioactive ceramic species, BGS-7 is composed of SiO2-CaO-P2O5-MgO-CaF2-B2O3. It forms an HA layer after about 24 h of insertion in the body fluid, which could provide a platform for creating a direct fusion with an adjacent bone. BGS-7 demonstrated a higher compressive strength, bending strength, and fracture toughness than HA as well as a larger contact area between the graft and bone than HA, titanium, and PEEK in a histological study. (Lee et al., 2010b). To minimize subsidence by distributing the stress, the geometry of the intervertebral spacer was changed to a right triangle-shaped valley. (Lee et al., 2013c). In the clinical application of posterior lumbar interbody fusion surgery, BGS-7 glass ceramic spacer and titanium cage revealed a similar fusion rates using CT scan at 12 months and clinical outcomes. (Lee et al., 2016).

In the present study, the BGS-7 spacer improved its durability without permitting any breakage of in the spacer. Neither breakage nor migration was found in this study. And also the follow-up period at least 60 months can conclusively prove the durability of the BGS-7 spacer, it is impressive to show no instrumental failure rate considering that cage breakage is relatively common problem in allograft spacer. Because all patient enrolled in this study had a plate augmentation, basic stability of the spacer can be further evaluated in the future study.

In the BGS-7 group, the average subsidence was 1.33 mm and the subsidence rate was 0%, while the average subsidence in the allograft spacer group was 2.27 mm and the subsidence rate was 26.6%, which was statistically significantly higher than BGS-7 spacer group. The subsidence rate in ACDF surgery with plate augmentation is reported as 9.6–55.6%. (Gercek et al., 2003; Song et al., 2010). In a meta-analysis that included 111 studies, the overall rate of subsidence was 31.4%. (Karikari et al., 2014). Depending on the type of interbody implant, the subsidence rate varied as follows; 26.9% for titanium, 22.8% for PEEK, 27.7% for fibular allograft, 35.9% for iliac autograft and 25.2% for carbon fiber-based cages. These results indicate that BGS-7 spacer is superior to other bone substitutes in terms of subsidence despite its high structural stability. Wu et al. reported that cage subsidence does not affect long-term clinical outcome and fusion rate, no patients with a spacer subsidence complained of any significant axial pain in this study. (Wu et al., 2012).

The long-term results of interbody fusion appeared to be acceptable. Eighteen of 18 patients who were followed for more than 60 months had enough fusion results with grades 4,5; and there were no patients with fusion grade 2, 3, rather few patients in allograft group showed grade 2, 3 fusion status. According to previous studies, the fusion rate seems to differ depending on the features of the space. (Chang et al., 2009). The use of autograft and plate augmentation was related to a higher fusion rate in previous studies. (Fraser and Hartl, 2007; Shriver et al., 2015). The overall fusion rate was reported as 89.5–97.4% (Fraser and Hartl, 2007) Mashhadinezhad et al. describes that the incidence of bony bridges in an autograft group was 16.6% at POD 3 months, 54% at POD 12 months. (Mashhadinezhad et al., 2014). Similarly, the fusion rate showed a tendency to increase over time in this study.

Pseudarthrosis is known to be associated with unfavorable outcome, including arm pain and axial pain. (Buttermann, 2017). Therefore, radiologic fusion status is one of the most important factors that influence clinical outcomes once enough decompression is obtained in ACDF surgery. Patients with radiologic evidence of pseudarthrosis also evaluated at long-term postoperative status, and smaller movements were observed at BGS-7 group, however, there were no statistical differences in distance of inter-spinous motion and fusion status. Those kind of interspinous motion parsimoniously related with long-term postoperative VAS, and BGS-7 isn’t inferior than allograft spacer. In our study, the favorable radiologic outcome appeared to result in acceptable prognosis.

### Limitations of study

At first, a limited number of enrolled patients are weak point of this study. More accumulated data are required although this study revealed the long-term clinical result of ACDF surgery with BGS-7 interbody fusion spacer. And stand-alone cage insertion without a plate augmentation might be better surgical technique to reveal the direct effect of spacer and maximize the difference from previously commercialized cervical spacer. Because this study was the first long-term clinical trial using BGS-7 glass ceramic spacer, a plate augmentation had to be applied for the safety of enrolled patients.

## Conclusion

BGS-7 spacer demonstrated reliability as a spacer used in ACDF surgery without instrumental failure. Although the high mechanical strength of the spacer, no subsidence was observed at over 60 months of the follow-up period, while significant subsidence was noted in 22.2% of the patients in the allograft group. Moreover, the early stabilization with a bony bridge formation was observed at the intermediate follow-up period, and the clinical outcomes were favorable consequently in the BGS-7 group more than 60 months after surgery without any adverse events. Therefore, the results suggested that theBGS-7 spacer is a safe and effective alternative to allograft for ACDF surgery.

## Data Availability

The data that support the findings of this study are available from the corresponding author upon reasonable request due to reasons of data sensitivity.
