# Supplementary material for: Long-term results comparison after anterior cervical discectomy with BGS-7 spacer (NOVOMAX®-C) and allograft spacer: A prospective observational study
Source: Front Bioeng Biotechnol. 2023 Apr 19;11:1100462. doi: 10.3389/fbioe.2023.1100462 (PMC10154694; doi:10.3389/fbioe.2023.1100462)

Supplementary figure 1.

The in vitro BGS-7 soaking test using simulated body fluid for up to 30 days. Images of BGS-7 at immediately after soaking (a-b) and after 30days of soaking (c-d). (a) cross sectioned, x500, (b) surface, x50000, (c) cross sectioned, x500, (d) surface, x50000,.


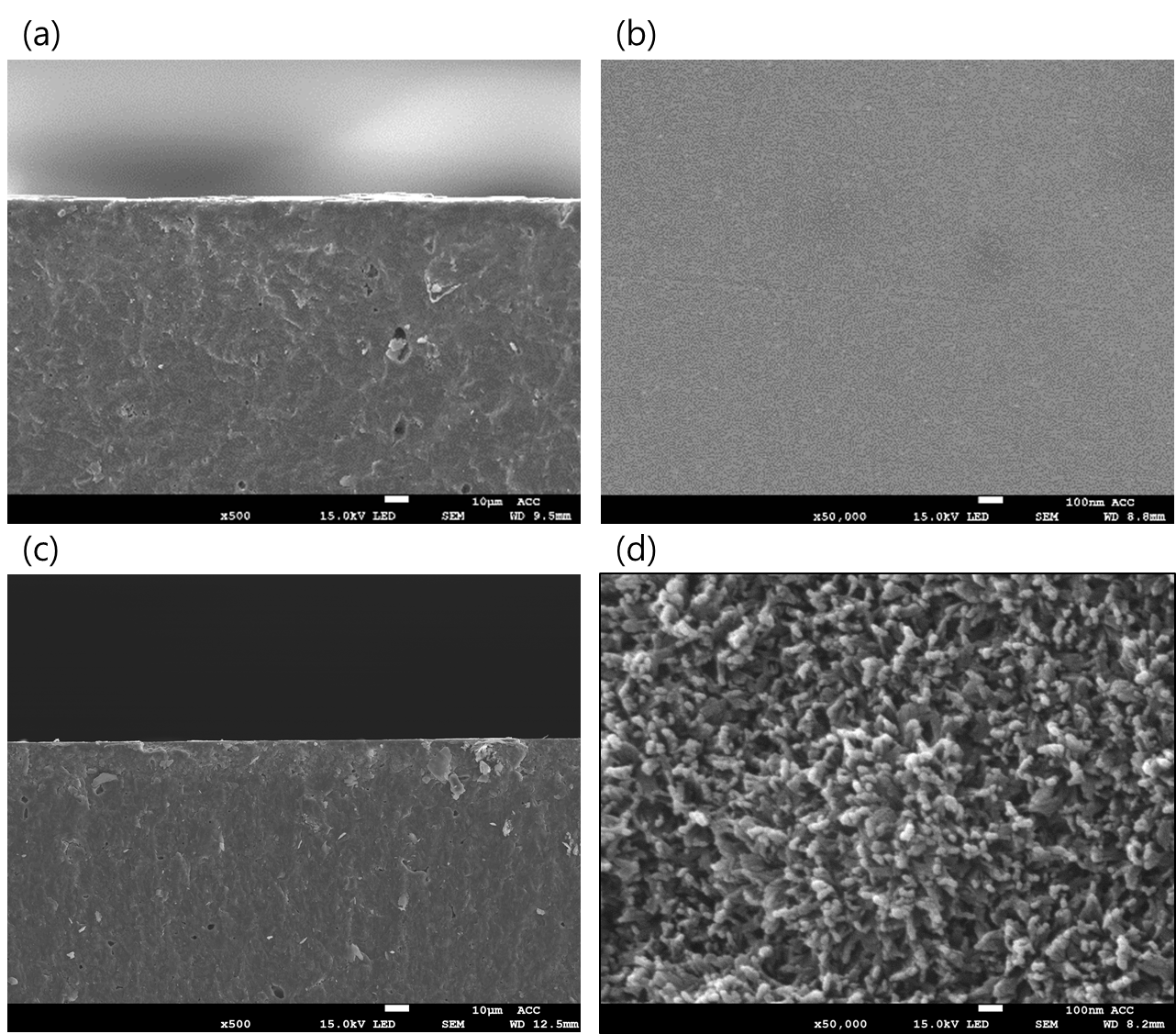

Supplement: Supplementary file 1 [file DataSheet1.docx]
